# Normal Metabolic Levels in Prefrontal Cortex in Euthymic Bipolar I Patients with and without Suicide Attempts

**DOI:** 10.1155/2015/165180

**Published:** 2015-05-13

**Authors:** Marlos Vasconcelos Rocha, Fabiana Nery-Fernandes, José Luiz Guimarães, Lucas de Castro Quarantini, Irismar Reis de Oliveira, Giovanna G. Ladeia-Rocha, Andrea Parolin Jackowski, César de Araujo Neto, Ângela Miranda-Scippa

**Affiliations:** ^1^Department of Neurosciences and Mental Health, Mood and Anxiety Disorders Program (CETHA), Universidade Federal da Bahia, 40110-905 Salvador, BA, Brazil; ^2^Image Memorial, Medicina Diagnóstica, Salvador, BA, Brazil; ^3^Laboratório Interdisciplinar de Neurociências Clínicas (LiNC), Universidade Federal de São Paulo, 04021-001 São Paulo, SP, Brazil; ^4^Department of Radiology, Universidade Federal da Bahia, 40110-905 Salvador, BA, Brazil; ^5^Postgraduate Program in Medicine and Health, Universidade Federal da Bahia, 40110-905 Salvador, BA, Brazil

## Abstract

*Introduction/Objective*. Evidence suggests that the prefrontal cortex has been implicated in the pathophysiology of bipolar disorder (BD), but few neurochemical studies have evaluated this region in bipolar patients and there is no information from BD suicide attempters using Proton Magnetic Resonance Spectroscopy (H^+^MRS). The objective was to evaluate the metabolic function of the medial orbital frontal cortex in euthymic BD type I suicide and nonsuicide attempters compared to healthy subjects by H^+^MRS. *Methods*. 40 euthymic bipolar I outpatients, 19 without and 21 with history of suicide attempt, and 22 healthy subjects were interviewed using the Structured Clinical Interview with the DSM-IV axis I, the Hamilton Depression Rating Scale, the Young Mania Rating Scale, and the Barratt Impulsiveness Scale-11 and underwent H^+^MRS. *Results*. We did not find any metabolic abnormality in medial orbital frontal regions of suicide and nonsuicide BD patients and BD patients as a group compared to healthy subjects. *Conclusions*. The combined chronic use of psychotropic drugs with neuroprotective or neurotrophic effects leading to a euthymic state for longer periods of time may improve neurometabolic function, at least measured by H^+^MRS, even in suicide attempters. Besides, these results may implicate mood dependent alterations in brain metabolic activity. However, more studies with larger sample sizes of this heterogeneous disorder are warranted to clarify these data.

## 1. Introduction

Bipolar disorder (BD) is a prevalent and chronic mental illness that is associated with high rates of suicidal behavior. The risk for suicide among BD patients is up to 20–30 times greater than that for the general population [1]. The cause of these high rates is unknown, but some studies have suggested a possible relationship between suicidal behavior with impulsivity [2, 3], and others have demonstrated an association of impulsivity and the orbital frontal cortex (OFC) dysfunction [4, 5].

In fact, evidence of an association between suicidal behavior and structural and functional abnormalities of the brain is mounting, and most of these regions involved in suicidal behavior are part of emotional regulation neuronal circuits, including amygdala, hippocampus, thalamus, and olfactocentric paralimbic cortices (insular, temporopolar and orbital frontal cortices) [6]. In this context, particularly, the OFC has strong connections with the amygdala, thalamus, and basal; in addition, the OFC mediate the individual's affect, impulse control, and recognition of reinforcing stimuli. In keeping with this line of reasoning, some authors have demonstrated an association between OFC dysfunction, decision-making impairment, and suicidal behavior; in a first study, they used the Iowa Gambling Task (IGT) and showed that violent suicide attempters make more disadvantageous choices than affective controls (patients without history of suicide attempt) [7]. In another study, these same authors compared suicide attempters and affective controls using an adapted version of the IGT during functional neuroimaging and showed that patients with history of suicide attempts (a) performed worse on a decision-making task, (b) had decreased activation in the OFC (and occipital cortex) for the contrast between disadvantageous and advantageous choices, and (c) had no difference for the contrast between wins and losses, which implies impairment of guiding of safe behavior [8]. Taken together, these findings suggest that suicide attempters were not able to evaluate risky choices appropriately. Interestingly, a component of impulsivity called “lack of premeditation” was associated to disadvantageous decision-making, according to some authors [9].

In addition, a link of evidence between metabolic dysfunction, prefrontal cortex, and suicidal behavior was the observation of serotonergic dysfunction from* in vivo* brain imaging study of nonfatal suicidal behavior using positron emission tomography (PET) scanning with 11-C-methyl-tryptophan (an analogue of tryptophan) that showed less uptake in medial orbital PFC in association with lethality of suicidal behavior [10] and another study found lower prefrontal cortical 5-HT2A binding in suicide attempters [11]. Besides, evidence from neuroimaging research has suggested the OFC as a crucial region involved in impulsive behavior; for instance, some authors have demonstrated a negative correlation between cortical thickness of the OFC and Barrat Impulsiveness Scale-11 (BIS-11) total and motor domain in healthy adults [12].

While a number of structural neuroimaging research techniques have been utilized to investigate a host of psychiatry disorders [13], Proton Magnetic Resonance Spectroscopy (H^+^MRS) is a noninvasive method that allows biochemical constituents to be directly assayed* in vivo*. Numerous studies utilizing this neuroimaging technique in BD have identified a number of metabolite changes in specific brain regions, such as the medial OFC, but most of these studies show mixed results, probably due to methodological issues, such as inclusion of patients with types I and II, in several phases of the illness, and medicated or drug-naïve patients [14–20]. Besides, information in terms of metabolic changes measured by H^+^MRS in BD suicide attempters is even scarcer.

To our knowledge, there are no previous studies comparing metabolic levels in frontal cortical gray matter measured by H^+^MRS in suicide and nonsuicide attempters in a homogeneous sample of type I euthymic BD outpatients.

So, the purpose of this study was to investigate metabolic levels of N-acetyl-aspartate (NAA), myo-inositol (mI), choline (Cho), and creatine (Cr) in the medial orbital prefrontal cortex in euthymic bipolar type I outpatients, with and without history of suicide attempts. We hypothesized that the neuronal metabolic function may be impaired in the cortical gray matter by H^+^MRS, even in the euthymic phase of the bipolar illness. In addition, we speculated that the patients with suicide attempts have more metabolic dysfunction in this area compared with the patients without history of suicidal behavior.

## 2. Methods

### 2.1. Participants

This study is part of a larger project of evaluation and treatment of patients with bipolar disorder treated at the research Center in Salvador-Bahia-Brazil (Mood and Anxiety Program of the Federal University of Bahia-CETHA), in which data is continuously collected. Patients were recruited from this center and were interviewed using the Structured Clinical Interview with the DSM-IV axis I (SCID-I) [21], the Hamilton Depression Rating Scale (HDRS) [22], the Young Mania Rating Scale (YMRS) [23], and the Barratt Impulsiveness Scale (BIS-11). The BIS is a self-report questionnaire composed of 30 items with Likert-type questions, rated from 1 (rarely/never) to 4 (almost always/always). Scoring yields a total score and 3 subscale scores derived by factor analysis: attention, motor, and nonplanning. Score varies from 30 to 120 and there is no established cut-off point [24]. The BIS differs from performance-based or cognitive measures of impulsivity as scores reflect self-rated behaviors rather than discrete cognitive processes and thus may be closer to psychiatric symptomatology. The euthymia criteria were scores for both the YMRS and HDRS below 7 points for at least two months. Demographic and clinical data were gathered through a questionnaire, and all assessment instruments were administered by two trained experts in psychiatry. Patients were classified as having positive suicidal history if they reported one or more self-injurious acts committed with intent to die.

Healthy controls were recruited among the patients' social network and were interviewed using the same evaluation instruments. The choice of these controls as a group was to try to prevent bias associated with differences in sociodemographic data between groups. None of these subjects had a current or past Axis I DSM-IV psychiatric disorder or a first-degree relative with an Axis I psychiatric disorder.

Exclusion criteria of all subjects were age less than 18 and more than 60 years, current serious medical conditions, history of head trauma and neurological disorders or substance abuse at any time, and current medical problems in the preceding six months.

### 2.2. Structural Magnetic Resonance Imaging HMRS Procedure

All MRI scans and H^+^MRS were acquired at the Image Memorial Clinic, Medicina Diagnóstica-Bahia-Brazil, using a 1.5-T Symphony Master/Class Siemens scanner (Ellagen, Germany) and conducted and interpreted in a blind manner by one research assistant (GLR), trained in H^+^MRS acquisition and spectral analysis and a second research assistant (MVR) confirmed in a blind manner the position of the voxel. The placement of the voxel was visually inspected to minimize white matter and CSF contents. To evaluate intrarater reproducibility, the same rater repeated the metabolites measurements twice, at least one week apart, for ten randomly selected subjects. Intrarater reliability was found to be high for all measurement (Kappa = 1,0; *P* = 0,0041).

The interpupillary line in comparison with transverse landmark light on the scanner was used to denote the position of each subject's head and T2-weighted images were obtained to localize the H^+^MRS voxel. To minimize head movements, the forehead was affixed with adhesive tape to the MRI stretcher and neck support was provided when necessary.

A single 8 cm^3^ voxel water-suppressed H^+^MRS was prescribed on the medial orbital prefrontal cortex, and spectra were acquired using point resolved spectroscopy sequence (TR = 1500 ms, TE = 30 ms, flip angle = 90°, NEX = 128). The most inferior of the gray matter voxel was positioned at a minimum level of 1.5 mm above the orbits and a maximum level of 3 mm.

### 2.3. Statistical Analysis

The data were entered into the Software Statistical Package for Social Sciences (SPSS) version 17.0, and the STATA statistical software package, version 11.0, was used in the statistical analysis. Associations were accepted when a *P* value was less than 5% (*P* value ≤ 0.05). The one-tailed test was used to ANCOVA and ANOVA, and for chi-squared test we used two-tailed test.

In the metabolites analyses the assumptions of normal distribution (One-Sample Kolmogorov-Smirnov Test) and equal variance around the required mean (Levene's Test of Equality of Error Variances) that tests the null hypothesis that the error variance of the dependent variable is equal across groups were satisfied by all the metabolites evaluated. To verify the possible differences in clinical and demographic characteristics between attempters, nonattempters, and healthy controls, chi-squared, Student's *t*-tests, Fisher's exact test, or univariate analysis of variance (ANOVA), with post hoc analysis (Bonferroni correction), were used, where appropriate. Only the length of illness did not have a normal distribution.

Lastly, the multivariate analysis (ANCOVA) model was used to compare the results of the metabolites between groups and adjusted for clinical and demographic variables. The statistical analysis of metabolites NAA, Cho, Cr, mI, NAA/Cr, Cho/Cr, and mI/Cr was done in two steps: step 1 compared nonsuicide attempters, suicide attempters, and healthy controls, and step 2 compared bipolar patients as a group and healthy controls.

The study was approved by the Local Medical Review Ethics Committee and was performed in accordance with the ethical standards of the Declaration of Helsinki. All subjects provided written informed consent prior to their inclusion in the study.

## 3. Results

We screened a total of 48 right-handed bipolar I outpatients and 8 were excluded, 5 because of a history of neurological illness or head trauma with loss of consciousness and 3 because they could not undergo an MRI exam. Twenty-five right-handed healthy controls were evaluated and 3 were excluded, 1 because of previous head trauma and 2 because they could not complete the MRI exam. Forty euthymic bipolar I patients, 19 with and 21 without a lifetime history of suicide attempt, and 22 healthy controls underwent the sociodemographics and clinical evaluation. During the subjects' assessments with MRI scan with H^+^MRS, 2 bipolar patients with suicide attempts, 2 bipolar patients without suicide attempts, and 6 healthy controls were excluded from the H^+^MRS analysis, because of the failure to meet spectral quality standards arising from susceptibility or movement artifacts.

Among attempters, 11 attempted once, 4 attempted twice, and 4 attempted three times; suicide attempt methods included overdose/poisoning, cutting, hanging, and jumping from heights. Finally, 5 patients adopted two different methods of suicide.

There were no significant differences between suicidal and nonsuicidal bipolar patients and healthy controls with respect to age, gender, and years of education (*P* > 0.5). There were also no significant differences between group of bipolar patients for age of onset, type of first episode, length of illness, history of psychiatric hospitalizations, number of psychiatric hospitalizations, lifetime psychoses, and family history of suicide or attempted suicide. However, the suicidal group had significantly more history of psychiatric comorbidities than nonsuicidal group (*P* = 0.03). All BD patients were on medication (mostly mood stabilizers).

Considering all selected patients and controls, suicide attempters showed higher mean scores than nonattempters and healthy controls in BIS total (67.3 ± 14.8; 58.3 ± 8.6; 58.5 ±9.0, resp.), BIS attentional (20.5 ± 3.9; 16.6 ± 2.5; 17.5 ± 3.4, resp.), BIS nonplanning (26.4 ± 6.2; 22.1 ± 4.9; 23.1 ± 4.4, resp.), and motor (20.4 ± 6.0; 19.5 ± 4.9; 17.9 ± 4.0, resp.) and the ANOVA with post hoc analysis (Bonferroni correction) revealed that the differences were significant in BIS total (*F* = 4.58, *P* = 0.01), BIS attentional (*F* = 7.75, *P* = 0.001), and BIS nonplanning (*F* = 4.44, *P* = 0.02). The BIS motor was not significant (*F* = 1.31; *P* = 0.28). All these clinical and demographic data have been described in a previous article published by our group [25].

Conversely, when only patients submitted to H^+^MRS scan are included in the BIS analysis, suicide attempters showed higher mean scores than nonattempters and healthy controls in BIS total (65.3 ± 14.7; 59 ± 8.8; 58.5 ± 9.6, resp.), BIS attentional (20.2 ± 4.0; 17.1 ± 2.5; 17.5 ± 3.7, resp.), and BIS nonplanning (25.8 ± 6.3; 22.1 ± 4.3; 22.5 ± 4.3, resp.), but the ANOVA with post hoc analysis (Bonferroni correction) revealed that only in attentional impulsivity the difference was significant (*F* = 4.09, *P* = 0.031). The BIS motor showed no significant lower mean score in suicide attempters compared to nonattempters and healthy controls (19.3 ± 5.8; 19.8 ± 4.9; 18.6 ± 4.4, resp.). As a group, BD patients also showed higher mean scores compared to healthy controls in BIS total (62 ± 12.18 versus 58 ± 9.64), BIS attentional (18.5 ± 3.62 versus 17.5 ± 3.68), BIS motor (19.5 ± 5.26 versus 18.5 ± 4.42), and BIS nonplanning (23.8 ± 5.6 versus 22.5 ± 4.33), but no significant difference was demonstrated (Table 1).

## 4. Spectral Analysis

There were no differences on H+MRS between BD suicide, nonsuicide attempters, and healthy controls (NAA: (*F* = 1.41; *P* = 0.252), mI: (*F* = 0.18; *P* = 0.829), Cho: (*F* = 0.26; *P* = 0.769), Cr: (*F* = 0.61; *P* = 0.543), NAA/Cr: (*F* = 0.19; *P* = 0.822), Cho/Cr: (*F* = 1.16; *P* = 0.320), and mI/Cr: (*F* = 0.07; *P* = 0.933)) (Table 2). In addition, we did not find any evidence of metabolic abnormality in BD patients as a group, compared to healthy subjects (NAA: (*F* = 2.13; *P* = 0.15), mI: (*F* = 0, *P* = 0.994), Cho: (*F* = 0.40; *P* = 0.527), Cr: (*F* = 0; *P* = 0.991), NAA/Cr: (*F* = 0.38; *P* = 0.536), Cho/Cr: (*F* = 1.27; *P* = 0.264), mI/Cr: (*F* = 0.13; *P* = 0.719)).

## 5. Discussion

The findings from the present study are in agreement with some papers that did not find differences in brain metabolite levels in the prefrontal cortex of bipolar patients compared to healthy controls [15, 26]. Also, these results could be explained by the use of the same methodology, such as only evaluating BD I patients under strict criteria of euthymia; assessed patients had been receiving lithium alone for a long time or associated with another psychiatric drug, including other mood stabilizers, antipsychotics, and antidepressants. Besides, gender and duration of illness of patients were also very similar to our sample. However, to our knowledge it is the first study that assessed the BD I group according to the history of attempted suicide and did not demonstrate differences on H^+^MRS between BD I suicide and nonsuicide attempters.

We speculated that these results may reflect mood-dependent alterations in brain metabolic activity, since studies that included both euthymic and noneuthymic patients have shown changes on metabolite spectra [14, 16, 17]. Another possibility is that the chronic use of psychotropic medical drugs may have a brain protective effect in this subgroup of patients. In fact, in our study, 30 patients (attempters and nonattempters, 75% of our sample) were receiving lithium, of which 21 were also receiving at least one psychiatric drug in addition to lithium. Ten patients were receiving an anticonvulsant associated or not with atypical antipsychotics. So, the combined chronic use of psychotropic drugs with neuroprotective or neurotrophic effects leading to a euthymic state for a longer period of time may improve neurometabolic function, at least where it is measured by H^+^MRS, even in a subgroup of patients with a severe characteristic, such as a history of suicide attempt.

In fact, according to our previous data, in our sample, 30.2% of the subjects were prescribed the first mood stabilizer in the year after the first affective episode (FMS ≤ 1), 22% after the first year before the 5th year (1 < FMS ≤ 5), and 47.8% 5 years after the first affective episode (FMS > 5). The lifetime prevalence of suicide attempts was 33.3% in the FMS ≤ 1 group, 32.2% in the 1 < FMS ≤ 5 group, and 58.6% in the FMS > 5 group. After adjusting for potential sociodemographic and clinical confounders in the two groups of patients with less than a 5-year delay for FMS (FMS ≤ 1 and 1 < FMS ≤ 5), the lower prevalence of suicide attempts remained significant when compared with the FMS > 5. When the FMS < 1 reference group was compared with the FMS > 5 group, adjusting for the same sociodemographic and clinical confounders, the higher prevalence of suicide attempts remained significant in the latter group, with a statistically significant difference between these groups [26]. These results support the protective clinical effect of the use of mood stabilizers on the suicidal behavior.

However, it is also important to highlight that in our sample the patients have low rates of psychiatric comorbidities (35%) compared to other studies that may contribute with a more favorable evaluation of the illness [27]. Additionally, the patients in the present study did not have current serious medical conditions, history of head trauma and neurological disorders or substance abuse at any time or current medical problems in the preceding six months, which may constitute a relatively benign clinical subgroup of BD I patients.

In general, the most important factor in controlling the neuroprogression of BD is the use of efficacious drugs combined with psychoeducation that may prevent relapses and recurrences of the illness [28, 29]. Keeping this issue in mind, mood stabilizers, such as lithium and valproic acid, used for the majority of patients in this study, are the most prominent drugs approved by the United States Federal Drug Administration (FDA) for treatment of BD [30]. Lithium and valproic acid, respectively, through inhibition of glycogen synthase kinase-3 (GSK-3) and the histone deacetylases (HDACs), regulate the transcription and expression of neurotrophic, angiogenic, and neuroprotective proteins, such as brain-derived neurotrophic factor (BDNF), glial cell-line derived neurotrophic factor (GDNF), and angiogenic vascular endothelial growth factor (VEGF). Also, lithium in particular acts on factors that affect apoptotic signaling, such as Bcl-2, p53, Bax, caspase, and heat shock proteins (HSP). Finally, lithium contributes to induction of the ubiquitin-proteasome system and autophagy, two major intracellular quality control mechanisms for protein clearance that prevents abnormal protein accumulation. Overall, these findings highlight the properties of lithium and probably other mood stabilizers to suppress cell death, attenuate neuroinflammation, and promote angiogenesis and cellular plasticity in BD patients [31], which contribute to the reduction of neuronal loss and consequently prevent the cognitive deficits in bipolar patients.

The positive effects of psychopharmacologic treatment on neural plasticity in BD patients may also be demonstrated from neuroimaging studies. In fact, some studies have shown an increase in gray matter volume in whole brain of BD patients treated with lithium [32], and untreated patients showed decreased left anterior cingulate volumes compared with either healthy controls or lithium-treated patients [33]. The NAA was also reported to be increased in the prefrontal cortex after lithium treatment [34]. These results may suggest evidence* in vivo* that the use of mood stabilizers, especially lithium, influences neurotrophic mechanisms in BD patients. Additionally, other authors who measured neuropsychological performance of BD patients in total remission under treatment with lithium as monotherapy demonstrated better performance on neuropsychological tests and higher plasma BDNF levels than BD patients with partial remission [35]. In summary, the benefits from optimal maintenance treatment regimens with lithium and probably other mood stabilizers go beyond the prevention of mood episodes and include neuroprotection, neural plasticity, and better functional and cognitive outcomes.

Other studies that have been conducted with BD patients compared to healthy subjects showed differences in the H^+^MRS scan, probably due to methodological issues, such as inclusion of euthymic and noneuthymic patients in different phases of the illness [14, 16, 17], assessment of more severe, hospitalized sample of patients and outpatients [14], inclusion of only medication-free patients at the time of the H^+^MRS scan [18], no stratification by age group (young versus old subjects) [36, 37], and inclusion of bipolar I and II patients [20], as previously described. Thus, these various methodological differences preclude the interpretation of results between studies. We think it likely that there are different subgroups of bipolar patients with different patterns of metabolic spectra, depending on the disease phenotype, since BD is a very heterogeneous illness with various clinical presentations.

There is also a debate regarding impulsivity as being linked to higher suicidal behavior among BD patients. Our study included BIS-11 as a tool for investigating trait impulsivity and in a previous paper of our group, it was shown that suicide attempters rated higher mean scores than nonattempters and healthy controls in BIS total, BIS attentional, and BIS nonplanning [25]. But, when we analyzed BIS data only from the patients and controls who submitted to H^+^MRS scan, the only significant difference was in attentional impulsivity; as a group, BD patients also showed higher mean scores compared to healthy controls in BIS total, BIS attentional, BIS motor, and BIS nonplanning, but no significant difference was demonstrated. These results from BIS data may be explained in different ways. The reduced sample size may limit the power of analysis, and although the patients in our study fulfilled strict criteria for euthymia, the data support previous findings of increased impulsivity in bipolar I patients as a trait, compared with healthy controls [38, 39]. In this context, more impulsive subjects may be less tolerant and restless, resulting in movement artifacts on H^+^MRS scan, a procedure that requires more time for the acquisition on spectral curve than on MRI scan. As a consequence, nonsignificant differences can emerge from the analysis by exclusion of these patients that could not conclude the H^+^MRS scan.

Therefore, in the present study, the only result that persists is the higher levels of BIS attentional in bipolar I patients with history of suicide attempt, compared with nonsuicidal and healthy controls. BIS attentional is thought to reflect a person's tendency to rapidly shift attention and a lack of cognitive persistence with the inability to tolerate cognitive complexity [40], and attention problems might predispose an individual to make rapid and inappropriate attributions and not to reappraise a potential conflict situation, increasing the likelihood of aggressive behavior, especially impulsive aggression [41]. In fact, some authors have demonstrated that impulsive aggression increases the risk of suicide behavior in patients with BD [42]. BIS nonplanning refers to a lack of consideration of future consequences, and its relationship with an inability to delay reward-related responses was demonstrated [43], which may reflect a sense of hopelessness about the future; in an event-related potential study, some authors have demonstrated a relationship between reduced P300 amplitude and suicide behavior and hopelessness but not with depressed mood [44]; a population-based, case-control study of nearly lethal suicide attempts found impulsive attempters with higher scores in Beck Hopelessness Scale than planned attempters and hopelessness, but not depression, distinguished impulsive attempters from the controls [45]. Ultimately, BIS motor is meant to measure the tendency of hasty or reckless action and it has been associated with manic symptoms [46]. Curiously, some authors have found a negative correlation between lethality of the attempts and motor impulsivity (i.e., higher lethality was associated with decreased motor impulsivity) [47]. In fact, it is important to highlight that in our second analysis of BIS, the motor impulsivity showed lower scores in the suicidal than in the nonsuicidal group. All these findings raise the complexity of research about impulsivity in BD patients.

Further complicating the relationship between impulsivity and suicidal behavior, a systematic review showed mixed evidence for an association between impulsivity and suicidal behavior from the studies; only 3 out of 11 studies found significant differences in overall impulsivity (BIS total scores) between attempters and nonattempters and no general consensus regarding association between BIS subscales and suicidal behavior was well established [48] and a recent meta-analysis was conducted for assessing the link between impulsivity and suicidal behavior; the authors have suggested impulsivity as a distal risk factor for suicide [49], reflecting the necessity for further investigation in this topic.

Although one of the strengths of our study is that it consisted of well-characterized euthymic BD I outpatients, without neurologic problems or other severe current medical comorbidities, with low rates of psychiatric disorders, which may result in a homogenous sample of BD patients, the results of the current study must be considered within the context of several limitations; first of all, the sample size of BD patients was reasonable for a study using a neuroimaging technique, but the number of BD was rather small, which leads to limitations in statistical power. Second, the single voxel technique requires a large voxel and limits the acquisition of small volumes of interest. Third, this study was conducted in an urban tertiary hospital serving a low- or middle-income population and these results may not be generalizable to other care services settings. Lastly, as described before, our sample was small and heterogeneous in terms of lethality and number of suicide attempts; so it was not possible to categorize according to frequency or lethality risk of suicidal behavior. Also, it was not possible to determine the lag time between suicide attempt and neuroimaging procedure as well.

## 6. Conclusion

Our results suggest that the brain metabolites measured by H^+^MRS are normal in the medial orbital frontal lobe in a subgroup of medicated BD I euthymic outpatients, suicide attempters or not, which may represent a phase-dependent metabolic profile or positive neurotrophic effects of the pharmacological treatment in this area. However, additional studies are needed on larger patient samples in order to clarify these issues.

## Figures and Tables

**Table 1:** Demographic and clinical data of the participants.

	BD I patients		Healthy controls	Statistics
	Suicidal (*n* = 19)	Nonsuicidal (*n* = 21)	(*n* = 22)	
Gender (*n*)				
Male	6	5	10	*x* ^2^ = 2.31, *P* = 0.31
Female	13	16	12	
Age, mean ± SD (years)	39.8 ± 11.4	42.0 ± 8.6	37.7 ± 13.5	*F* = 0.75, *P* = 0.47
Educational level, mean ± SD (years)	12.0 ± 3.0	11.2 ± 3.7	11.2 ± 2.7	*F* = 0.37, *P* = 0.69
Age of onset, mean ± SD (years)	24.3 ± 9.0	25.3 ± 9.4	NA	*t* = 0.34, *P* = 0.73
Length of illness, mean ± SD (years)	15.6 ± 7.2	16.5 ± 10.7	NA	*t* = 0.31, *P* = 0.75
Hospitalizations (*n*)				
Yes	13	18	NA	*x* ^2^ = 1.71, *P* = 0.26
No	6	3	NA	
Number of hospitalizations, mean ± SD	6.0 ± 5.8	3.1 ± 2.8	NA	*t* = −1.80, *P* = 0.08
Type of first episode				
Depression	11	10	NA	*x* ^2^ = 1.84, *P* = 0.39
Mania	7	11	NA	
Unknown	1	0	NA	
Lifetime psychoses (*n*)				
Yes	15	9	NA	*x* ^2^ = 2.77, *P* = 0.96
No	4	11	NA	
Family history of suicide (*n*)				
Yes	6	4	NA	*x* ^2^ = 0.83, *P* = 0.47
No	13	17	NA	
Family history of attempt suicide (*n*)				
Yes	2	4	NA	*x* ^2^ = 0.56, *P* = 0.66
No	17	17	NA	
Psychiatric comorbidities (*n*)				
Yes	10	4	NA	*x* ^2^ = 4,51, *P* = 0.03^*^
No	9	16	NA	
^†^BIS total score, mean ± SD	65.3 ± 14.7	59 ± 8.8	58,5 ± 9,6	*F* = 1.88, *P* = 0.312
Attention score, mean ± SD	20.2 ± 4.0	17.1 ± 2.5	17.5 ± 3.7	*F* = 4.09, *P* = 0.031^*^
Motor score, mean ± SD	19.3 ± 5.8	19.8 ± 4.9	18.6 ± 4.4	*F* = 0.27, *P* = 0.28
Nonplanning score, mean ± SD	25.8 ± 6.3	22.1 ± 4.3	22.5 ± 4.3	*F* = 2.81, *P* = 1

^†^Only from the patients submitted to H^+^MRS.

^*^Significant at the 0.05 level (2-tailed).

NA—no applicable.

**Table 2:** Medial orbital prefrontal cortex metabolic levels between bipolar patients with and without suicide attempt and healthy controls (mmol/l ± SD).

Metabolic levels	BD I patients		HC	Statistics
	Suicidal *n* = 17 mean (SD)	Nonsuicidal *n* = 19 mean (SD)	*n* = 16 mean (SD)	
NAA	2.79 ± 0.58	2.95 ± 0.58	3.12 ± 0.54	*F* = 1.41, *P* = 0.252
Myo-inositol	0.88 ± 0.42	0.97 ± 0.42	0.93 ± 0.41	*F* = 0.18, *P* = 0.829
Choline	1.32 ± 0.37	1.36 ± 0.42	1.27 ± 0.31	*F* = 0.26, *P* = 0.769
Creatine	1.53 ± 0.39	1.70 ± 0.53	1.63 ± 0.43	*F* = 0.61, *P* = 0.543
NAA/creatine	1.86 ± 0.36	1.85 ± 0.39	1.93 ± 0.51	*F* = 0.19, *P* = 0.822
Choline/creatine	0.86 ± 0.17	0.81 ± 0.14	0.78 ± 0.11	*F* = 1.16, *P* = 0.320
Myo-inositol/creatine	0.61 ± 0.31	0.63 ± 0.35	0.59 ± 0.27	*F* = 0.07, *P* = 0.933
Choline	2.79 ± 0.58	2.95 ± 0.58	3.12 ± 0.54	*F* = 1.41, *P* = 0.252
Creatine	0.88 ± 0.42	0.97 ± 0.42	0.93 ± 0.41	*F* = 0.18, *P* = 0.829

Significant at the 0.05 level (2-tailed).

Degrees of freedom = 1.

NAA: N-acetyl-aspartate.

HC: Healthy controls.
